# Analysis of European Association of Urology Guidelines 2023 and its adherence to GRADE methodology

**DOI:** 10.1002/gin2.70004

**Published:** 2024-10-09

**Authors:** Philipp Dahm, Mathew Holten, John P. Ratanawong, Andrew Lauwagie, Daniel A. Gonzalez‐Padilla

**Affiliations:** ^1^ Division of Urology Minneapolis VAMC Minneapolis Minnesota USA; ^2^ Department of Urology University of Minnesota Minneapolis Minnesota USA; ^3^ University of Minnesota Medical School Minneapolis Minnesota USA; ^4^ Department of Urology Wayne State University Detroit Michigan USA; ^5^ Department of Urology Clínica Universidad de Navarra Madrid Spain

**Keywords:** clinical practice guidelines, evidence‐based medicine, GRADE, systematic reviews

## Abstract

**Introduction:**

The European Association of Urology (EAU) has reported the use of a modified Grading of Recommendations Assessment, Development, and Evaluation (GRADE) approach for developing guideline recommendations since 2018; however, the full adoption of GRADE remains a work in progress.

**Methods:**

Two members of the research team identified and independently abstracted all individual recommendations and summary of evidence statements available from all EAU guidelines published in 2023. We evaluated the current adherence to established criteria for appropriate GRADE use, the proportion of strong and weak recommendations, the underlying strength of evidence, and criteria for appropriate use of GRADE.

**Results:**

We included a total of 20 guideline documents with a total number of 1985 recommendations. The median number of strong recommendations was 46 (interquartile range: 32–87), representing 62.6% of all recommendations. The surgical thromboprophylaxis guideline had the lowest percentage of strong recommendations (18.2%; 30/165), and the neuro‐urology guideline had the highest percentage (95.5%; 42/44). EAU guidelines provided 2580 summaries of evidence statements, of which 1608 reported a level of evidence. The reported levels of evidence were I, II, III and IV/V at 31.8%, 28.5%, 32.4% and 7.3%, respectively. Of the six criteria for the appropriate use of GRADE, criterion 6, which reports the framing of recommendations as strong or weak, was met consistently; all other criteria were met only by a single guideline on thromboprophylaxis.

**Conclusions:**

The EAU guidelines adoption of GRADE has been largely limited to the framing of recommendations as strong or weak. In all but one guideline, published guidelines did not meet any of the other criteria for appropriate GRADE use. Increased efforts to fully adopt GRADE, similar to its implementation in the thromboprophylaxis guideline in future editions, may enhance trust and uptake of EAU guidelines, thereby assisting policymakers and improving patient care.

## Highlights

- The EAU guidelines use GRADE for categorizing recommendations as ‘strong’ or ‘weak’, but lack full integration of key GRADE criteria.
- 63% of the 1985 recommendations were strong, though many were based on low‐quality evidence.
- Only the thromboprophylaxis guideline fully adhered to all six GRADE criteria.

## 1 INTRODUCTION

Clinical practice guidelines (CPGs) have been defined as ‘systematically developed statements to assist practitioner and patient decisions about appropriate healthcare for specific clinical circumstances’.^1^ They are integral to both evidence‐based clinical practice and informing health policy and should be developed using a transparent and methodologically rigorous framework that meets the criteria set forward by the National Academy of Medicine.^2^ In pursuit of this standard, more than 110 governmental and professional organizations across 19 countries have adopted the Grading of Recommendations Assessment, Development, and Evaluation (GRADE) approach.^3^ GRADE uses the categories of strong and weak (or conditional) recommendations and rates the underlying certainty of evidence (formerly quality or level of evidence) as high, moderate, low, or very low.^4^ Furthermore, it describes additional domains (such as values and preferences, balance of benefits to harms, and resource utilization) for guideline panel members to explicitly consider when deliberating over recommendations. GRADE has also described a structured process to facilitate the translation of the available evidence into usable recommendations to foster appropriate stakeholder representation and transparency.^5^, ^6^

The European Association of Urology (EAU) and the American Urological Association (AUA) are the largest urological associations worldwide that publish widely accepted, authoritative guidelines that influence patient care, health policy, and coverage determinations by payors. The EAU published its first guideline in 2000^7^ and reported the use of the GRADE approach for creating guidelines in 2014. Since 2018, it has adopted the nomenclature of strong and weak recommendations.^8^ The EAU's commitment to the GRADE methodology was reaffirmed in the two most recent versions of their *Guidelines Office Development Handbook* (versions July 2017 and March 2022).^9^ We performed this analysis to evaluate the extent to which current EAU guidelines follow published criteria for applying or using GRADE.^3^, ^10^, ^11^

## 2 METHODS

As an extension of prior work published as a commentary,^12^ we identified all EAU guidelines available from the Uroweb website (date of search: 6 January 2024) in portable document format. Two reviewers independently abstracted the title, the number of recommendations (and whether ‘strong’ or ‘weak’), and certainty of evidence (reported as strength of evidence using a modified level of evidence system by the Oxford Center for Evidence‐Based Medicine) or the GRADE categories (‘high’, ‘moderate’, ‘low’ or ‘very low’) from each guideline. We assessed adherence to the appropriate use of GRADE for both evidence synthesis and guidance documents as provided by the GRADE Working Group website.^3^ These include six domains: (1) definition of certainty of evidence consistent with GRADE definitions, (2) explicit consideration of the GRADE domains for rating the certainty of evidence, (3) certainty of evidence rating using three or four categories (such as high, moderate, low and/or very low), (4) evidence summaries and evidence‐to‐decision criteria as the basis of judgments regarding the strength of recommendations, (5) explicit consideration of the GRADE criteria for determining the direction and strength of a recommendation and (6) assessment of strength of recommendations using two categories with appropriate definitions for each category (such as strong and weak/conditional). Additionally, we retrieved all supplementary documents cited in a given guideline and examined the methodologies described in the EAU Guidelines Office Development Handbook (Version July 2017 and March 2022).^9^ The data abstraction and coding were performed independently, and in duplicate by two members of the research team (MH and AL), with input from a third investigator (PD) as needed.

We performed descriptive statistics to report measures of central tendency and distribution using SPSS version 28. We did not perform statistical hypothesis testing. No formal statistical testing was performed to assess interobserver agreement which was consistently very good.

## 3 RESULTS

**Figure 1:**
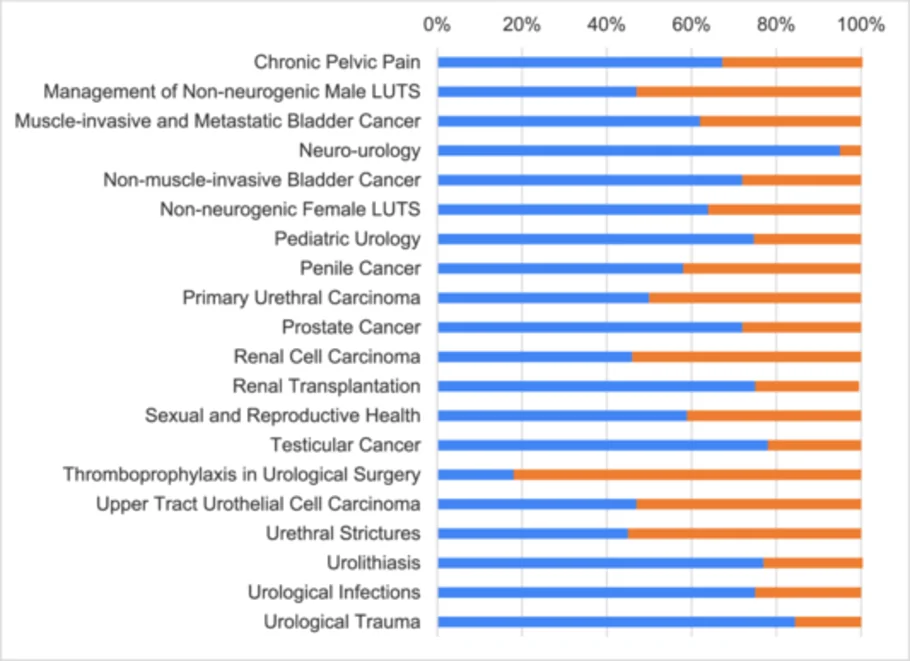
Proportion of strong (blue) and weak (orange) recommendations per guideline. Most guidelines have more than half of their recommendations graded as strong.

We identified a total of 20 EAU CPGs, all of which were included in this study. The 20 included guidelines issued a total of 1985 recommendations, all categorized as either strong (*n* = 1243; 63%) or weak (*n* = 742; 37%). The number of recommendations per guideline ranged from 32 (Upper Urinary Tract Urothelial Cell Carcinoma) to 237 (Sexual and Reproductive Health). Regarding strong recommendations, the median number per EAU guideline was 46 [interquartile range (IQR): 32; 87; Figure 1] with a mean percentage of 63%. The range extended from 18% (30/165; thromboprophylaxis) to 95% (42/44; neuro‐urology).

The majority of EAU guideline recommendation statements were not reported with explicitly linked summaries of evidence statements; instead, they were presented in independent tables. Furthermore, the number of guideline recommendations did not correspond to the number of evidence statements. Some guidelines had considerably more recommendations than evidence statements, or vice versa. These discrepancies in the ratio of recommendation‐to‐evidence statements were most apparent within the guideline on Prostate Cancer, which had 154 more recommendations than evidence statements (175 and 21, respectively). Out of the 20 guidelines assessed, only the guideline on Thromboprophylaxis used GRADE certainty of evidence ratings, in which there was a one‐to‐one ratio between certainty of evidence and corresponding recommendation statements.

**Figure 2:**
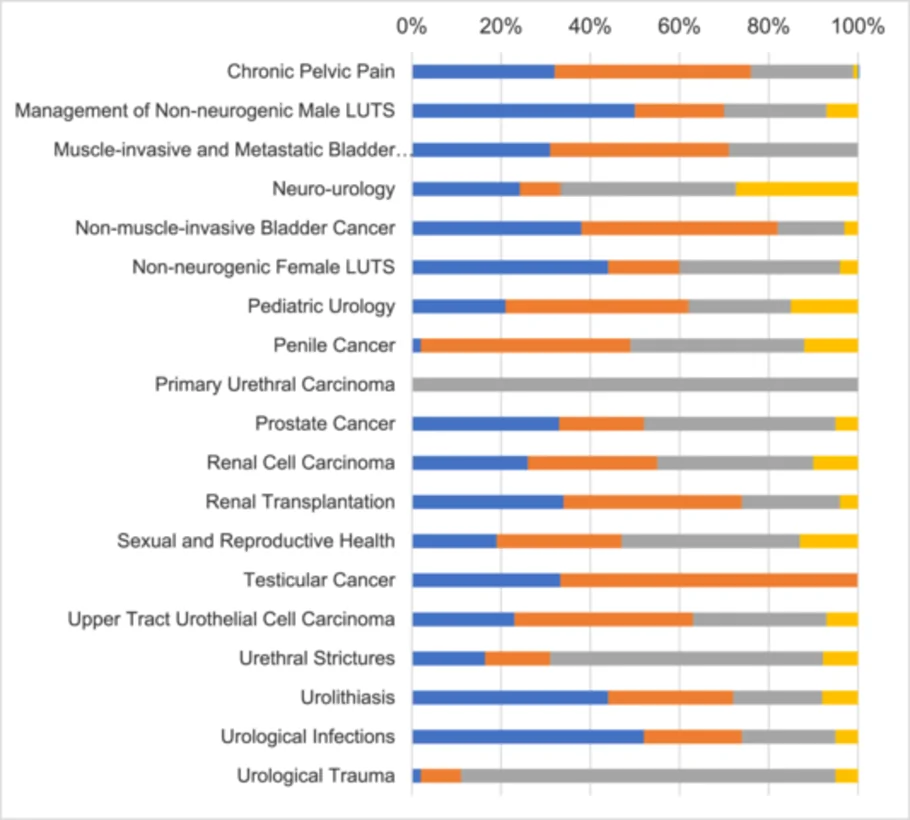
Proportion of ‘level of evidence’ backing summary of evidence statements; blue, Level I; orange, Level II; grey, Level III and yellow, Level IV or V.

In total, there were 2580 summaries of evidence statements reported as levels of evidence, categorized as I, II, III and IV/V at 31.8%, 28.5%, 32.4% and 7.3%, respectively (Figure 2). Overall, we observed the highest number of Level I evidence statements within the guideline for Nonneurogenic Female Lower Urinary Tract Symptoms (LUTS) (*n* = 114), whereas Primary Urethral Carcinoma had the least (*n* = 0). The Paediatric Urology guideline had the highest number of level IV/V evidence statements (*n* = 26), whereas the lowest numbers were found in the Primary Urethral Carcinoma, Testicular Cancer, Thromboprophylaxis, and Muscle‐invasive and Metastatic Bladder Cancer guidelines (*n* = 0).

Of the 20 EAU guideline documents analyzed, only the Thromboprophylaxis guideline met criteria 1–5 of the six criteria in accordance with the GRADE methodology. Aside from this guideline, none of the other 19 EAU guidelines defined the certainty of evidence consistent with GRADE (criterion 1), opting to utilize a modified level of evidence framework instead. This approach did not consider each of the established GRADE domains for assessing the certainty of evidence (criterion 2). Likewise, the guidelines did not categorize the overall certainty of evidence using three or four categories (such as high, moderate, low, and/or very low; criterion 3) and instead utilized levels of evidence from I to V that were defined differently. None of the EAU guidelines provided evidence summaries specific to individual recommendations (criterion 4) or specified the criteria that informed the direction (for vs. against) or strength (strong vs. weak) of recommendations (criterion 5). Despite this, all 20 guideline documents appropriately framed their recommendations using two categories (strong or weak) and defined these in a manner consistent with GRADE (criterion 6).

## 4 DISCUSSION

In this study, we found that the current 2023 EAU guidelines consistently applied the GRADE framework for how clinical recommendations are framed, namely as ‘strong’ or ‘weak’ recommendations. Furthermore, the EAU provided specific, GRADE‐appropriate guidance on how to interpret and utilize these recommendations for patients, physicians, and policymakers. However, EAU guidelines failed to adhere to GRADE guidance regarding other criteria of appropriate GRADE use. Namely, EAU guidelines used modified ‘levels of evidence’ of the Oxford Center of Evidence‐Based Medicine to describe the certainty of evidence, did not provide explicitly linked evidence profiles with each recommendation, and were also not transparent on how panel members reached consensus on a specific recommendation or what domains they considered (such as relationship of anticipated benefit to harm, patients' values, and preferences, and expected resource utilization). Lastly, we observed that a large proportion of recommendation statements were categorized as strong, which is unusual and also suggests that GRADE guidance was not followed appropriately.

This study represents a critical external evaluation of the EAU Guideline Office's implementation of the GRADE approach. The strengths of this study lie in its comprehensive scope, encompassing all currently published EAU guideline documents as of January 2024. All steps of the data abstraction and coding process were performed in duplicate by two independent members of the investigative team, which was led by individuals with substantial expertise in guideline development and GRADE methodology. Potential limitations relate to the following issues: First, the scope of our study was specific and narrow, focusing on the EAU guidelines' application of the GRADE approach. We did not evaluate other important aspects of the guideline development process, such as the degree of stakeholder involvement or editorial independence, nor did we evaluate the trustworthiness or the overall quality of the EAU guidelines, which could be assessed by the Appraisal of Guidelines for Research and Evaluation (AGREE) II tool.^13^ Additionally, we did not evaluate the overall transparency of reporting using the Reporting Items for Practice Guidelines in Healthcare (RIGHT) checklist.^14^ Second, our analysis relied on publicly available documents, namely the full guideline documents as well as the EAU guidelines office development handbook,^9^ to inform the actual processes followed by individual guideline panels. While it is plausible that EAU guideline panel deliberations followed a formal evidence‐to‐decision framework, such important information should have been reported. Lastly, we acknowledge the thromboprophylaxis in urological surgery guideline,^15^ which notably met all criteria for the appropriate use of GRADE,^16^ thereby providing a positive example of successful GRADE implementation.

Several prior studies have assessed the methodology and transparency of reporting urological guidelines,^17^, ^18^, ^19^ using the above‐mentioned AGREE,^20^ AGREE‐II^13^ or RIGHT checklists.^14^ However, our study distinguishes itself by focusing specifically on the appropriate application of GRADE in the EAU guidelines, making it comparable to a study by Dixon et al. In their study, Dixon et al. evaluated all evidence‐based guidelines from professional organizations based in the United States that were listed by the National Guideline Clearing House and self‐reported the use of GRADE.^21^ Of 67 guidance documents analyzed, the majority were related to internal medicine (74.6%), with only a small percentage (16.4%) originating from surgical sub‐specialities (trauma, vascular, colorectal and orthopaedics) and none were urology‐related. Regarding compliance with criteria for the appropriate use of GRADE, most (74.6%) assessed the strength of recommendation in two categories, similar to the EAU guidelines. A majority (89.6%) also defined the certainty of evidence consistent with GRADE, a practice not observed in the EAU guidelines. However, only a few (13.4%) explicitly referenced all relevant domains for defining the certainty of evidence, and only approximately half (53.7%) provided evidence profiles to underpin each recommendation, items that were found in very few (3.8%) EAU guideline statements. The findings of this study suggest that other guideline developers also face challenges in the application of GRADE, although many appear to achieve a greater degree of compliance.

Two recent and related studies have examined the methodology of the AUA, the other main producer of guidance for the speciality of urology.^22^, ^23^ Peña et al. assessed to what extent existing systematic reviews were cited by AUA guidelines published between 2004 and 2020, as well as their methodological quality, using the Measurement Tool to Assess Systematic Reviews (AMSTAR)‐2 instrument.^22^ They found that among the 18 included guidelines, 81 systematic reviews addressing focused clinical questions were cited. The methodological quality of these reviews was rated as critically low, low, moderate, and high in 47%, 24.1%, 27.7% and 1.1% of cases, respectively. In their conclusions, the authors emphasized the necessity to improve the quality of systematic reviews underpinning urological guidelines. Previous studies have consistently demonstrated that the methodological quality of systematic reviews published in the urological literature is generally low,^24^, ^25^, ^26^, ^27^ suggesting that this issue extends beyond guidelines. A study by Cleveland et al. focused on the type and wording of AUA recommendations.^23^ The authors identified 18 published AUA guideline documents that provided a median of 36 recommendations (IQR: 29–48). Of these recommendations, nearly half (349; 46.3%) were not evidence‐based and were classified as ‘expert opinion’ (25.6%) or ‘clinical principle’ (20.7%). Meanwhile, 17.9%, 24.8% and 11% were categorized as ‘strong’, ‘moderate’ or “conditional” recommendations, respectively. One significant concern with the AUA guideline strength of recommendation framework is the lack of guidance to users regarding the practical implications of this five‐tiered system, thereby undermining the meaning of the recommendation type the panel selects. Meanwhile, the GRADE approach used by the EAU clearly operationalizes how patients, health providers, and policymakers should interpret guideline statements depending on whether they are ‘strong’ or ‘weak/conditional’.

A further notable finding of this study was the predominance of recommendations being categorized as ‘strong’. According to published GRADE guidance,^10^ strong recommendations signal to patients and clinicians that ‘all or almost all informed people would make the recommended choice for or against an intervention’. A strong recommendation signals that the panel perceives there to be a clear imbalance of desirable versus undesirable consequences.^11^ In contrast, weak recommendations imply that clinicians devote considerable time in the shared decision‐making process to ensure that the choice made reflects individual values and preferences, a less critical factor for strong recommendations. Therefore, the prevalence of strong recommendations for approximately two‐thirds of its recommendations carries important implications for patient counselling. The finding that nearly 4 in 10 strong recommendations were based on evidence categorized as level 3 or 4/5 further raises the question as to whether a strong recommendation was indeed appropriate, given GRADE guidance indicates their use be mostly restricted to settings with high or moderate certainty of evidence.^11^ Prior studies have analyzed this phenomenon of incongruent recommendations (strong recommendations despite low or very low certainty evidence).^28^, ^29^, ^30^ For example, Brito et al. found that 58% of recommendations by the Endocrine Society were categorized as ‘strong’, of which 59% were based on low or very low certainty of evidence.^28^ Similarly, Alexander et al. found that approximately half of World Health Organization recommendations were strong, with half of these based on low or very low certainty of evidence. In a subsequent study, the same authors identified the main reasons for discordant recommendations, including panel members' scepticism about the value of conditional recommendations, concerns about their potential neglect, perceived high certainty of benefits (despite assessing the certainty as low), and political considerations.^31^ Yao et al. studied discordant recommendations by the American College of Cardiology/American Heart Association and the American Society of Clinical Oncology, finding that recommendations labelled as ‘consensus‐based’ were more likely to produce discordant recommendations than those characterized as ‘evidence‐based’.^30^ In summary, these studies suggest that the issue of discordant recommendations is not unique to EAU guidelines. Proposed strategies to address this issue include selecting panellists committed to GRADE principles, providing additional training for panellists and implementing formal processes that promote adherence to GRADE.^31^

The findings of this study highlight the significant challenges faced by organizations such as the EAU in adopting GRADE. Although GRADE is freely accessible, its effective use requires specific training and expertise among supporting methodologists and panel participants.^32^ Despite the increasing publication of systematic reviews, they often exhibit poor methodological quality and commonly do not use the GRADE approach to assess the certainty of evidence.^27^ This represents an important reason why many guideline developers choose to rely on their own systematic reviews (as practiced by the EAU) or commission specialized agencies (such as Evidence‐Based Practice Centers in the United States) to perform this work specifically for a given guideline (as does the AUA).

It appears important to note that the challenges faced by the EAU on its path toward rigorous and transparent guidelines meeting the highest standards are not unique to urology but are shared by many professional organizations.^21^, ^33^ The existence of the EAU guideline on perioperative thromboprophylaxis proves that is possible to implement all aspects of the GRADE approach. One important strategy may be to prioritize the updating of certain guideline topics, recognizing that different areas of clinical research may advance at varying paces, with new waves of evidence arising at different times. Instead, rather than relying on a fixed timetable for producing updates, guidelines should be ‘living’ documents where updates are triggered by substantial additions to the evidence base which may result in new/modified recommendations. Similarly, ‘living’ systematic reviews that utilize automation for literature searches and data abstraction (powered by artificial intelligence) may help enact such updates ‘as needed’ instead of adhering to a fixed schedule. Partnering with other organizations invested in high‐quality evidence synthesis, such as Cochrane, may further reduce the overall burden and permit the redistribution of scarce resources to areas other than evidence synthesis.

## 5 CONCLUSION

The EAU demonstrates a commitment to employing GRADE in its approach to developing evidence‐based recommendations. Their guidelines consistently use the GRADE framework in formulating ‘strong’ and ‘weak’ recommendations, thereby providing explicit guidance for patients, providers, and policymakers alike. However, increased efforts are necessary for EAU guideline panels to adopt other critical aspects of the GRADE approach, including the associated rating of the certainty of evidence, while also increasing transparency in the evidence‐to‐recommendation process. Full adoption of GRADE is expected to foster increased trust and uptake of the guidelines, enhance opportunities for collaboration, support evidence‐based policymaking, and ultimately improve patient care.

## AUTHOR CONTRIBUTIONS

**Philipp Dahm, Daniel A. Gonzalez‐Padilla**: Conceptualization. **Philipp Dahm, Mathew Holten, John P. Ratanawong, Andrew Lauwagie, Daniel A. Gonzalez‐Padilla**: Data curation. **Philipp Dahm**: Formal analysis. **Philipp Dahm, Mathew Holten, John P. Ratanawong, Andrew Lauwagie, Daniel A. Gonzalez‐Padilla**: Investigation. **Philipp Dahm, Daniel A. Gonzalez‐Padilla**: Methodology. **Philipp Dahm, Daniel A. Gonzalez‐Padilla**: Project administration. **Philipp Dahm**: Supervisión. **Philipp Dahm, Mathew Holten, John P. Ratanawong, Andrew Lauwagie, Daniel A. Gonzalez‐Padilla**: Validation. **Philipp Dahm, Mathew Holten, John P. Ratanawong, Andrew Lauwagie, Daniel A. Gonzalez‐Padilla**: Writing—original draft preparation. **Philipp Dahm, Daniel A. Gonzalez‐Padilla**: Writing—review & editing.

## CONFLICT OF INTEREST STATEMENT

The authors declare no conflict of interest.

## ETHICS STATEMENT

This article did not involve any human research as it is an external analysis of guidelines; hence, the ethics department was involved.

## Data Availability

The data that support the findings of this study are openly available in EAU Guideline Repository at https://uroweb.org/guidelines.
